# Platelet apheresis with additive solution and plasma rinseback affects the cellular composition of LRS chamber products

**DOI:** 10.1038/s41598-025-04350-4

**Published:** 2025-06-06

**Authors:** Katharina Kronenberg, Frauke Dormann, Andreas Brosig, Irene Pamler, Edward K. Geissler, Ralph Burkhardt, Robert Offner, James A. Hutchinson, Viola Haehnel

**Affiliations:** 1 Department of Surgery, University Hospital Regensburg, Franz-Josef-Strauß-Allee 11, 93053 Regensburg, Germany; 2 Institute of Clinical Chemistry and Laboratory Medicine, Transfusion Medicine, University Hospital Regensburg, Regensburg, Germany

## Abstract

Human leukocyte concentrates recovered from leukocyte reduction system (LRS) chambers of the Trima Accel automated blood collection device are a by-product of platelet apheresis that is often used in research. The Trima Accel software was obligatorily updated in 2023 from version 6 to 7. Here, we investigated software-dependent differences in the cellular composition of LRS concentrates when performing apheresis with either plasma or platelet additive solution. When using plasma as suspension medium, the software update to Trima 7 led to higher fraction of B cells as revealed by flow cytometry. Compared to platelets-in-plasma collection, the total recovered volume and leukocyte density was significantly reduced when running Trima 7 software with platelet additive solution including a plasma rinseback. Moreover, the proportion of lymphocytes and monocytes in these products was lower, whereas the proportion of neutrophils and eosinophils was higher. Researchers working with leukocytes isolated from LRS chambers should be aware that performing apheresis with platelet additive solution alters the composition of their starting material, which could affect the interpretation of some experiments.

**Supplementary Information:**

The online version contains supplementary material available at 10.1038/s41598-025-04350-4.

## Introduction

**Fig. 1:**
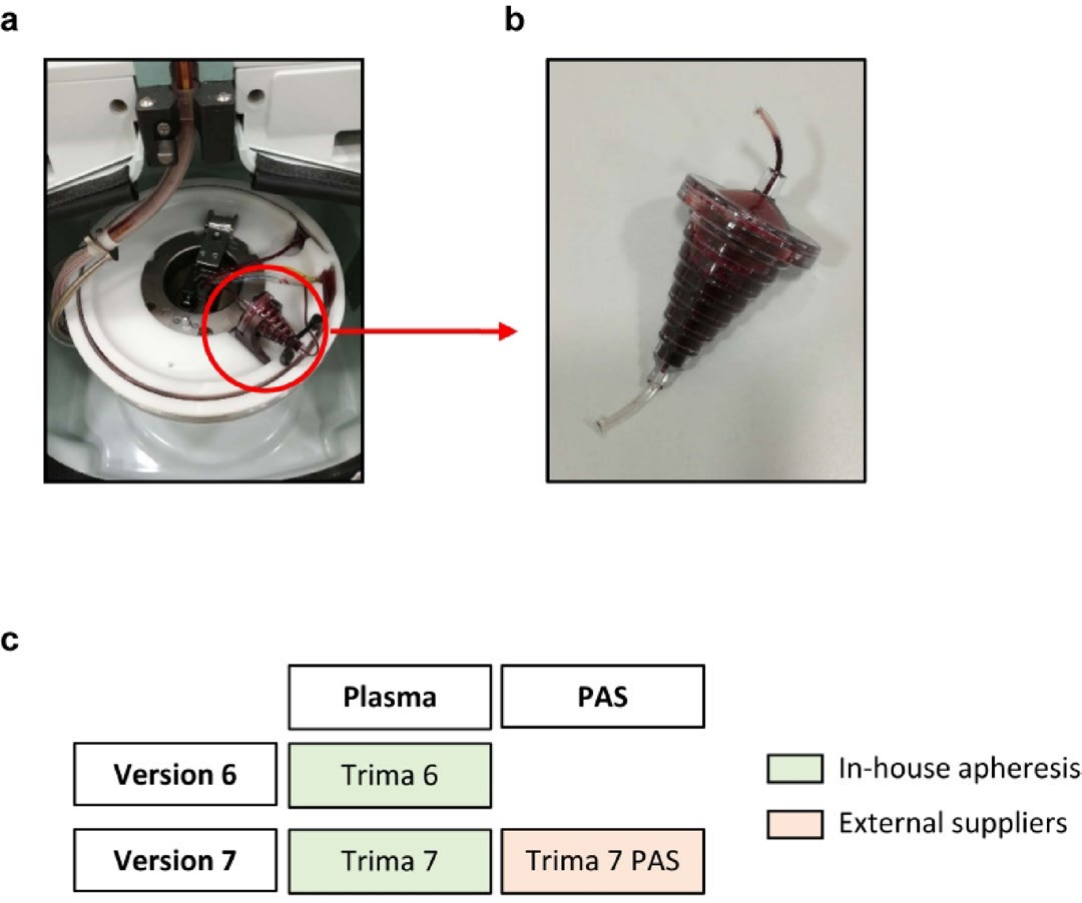
LRS chamber products were obtained from platelet apheresis using Trima Accel software version 6 and 7. In-house platelet apheresis were run using the Trima Accel automated blood collection system including the Trima Accel LRS Platelet Plasma Set (**a**). The LRS chamber (**b**) as a source of highly concentrated leukocytes was collected after apheresis and the composition of the cell product was analyzed. Currently, a software update from Trima Accel version 6 (Trima 6) to version 7 (Trima 7) was available, both using plasma for platelet collection (**c**). LRS chamber products generated during apheresis with software version 7 and platelet additive solution (PAS) instead of plasma (Trima 7 PAS) were obtained from external suppliers.

Freshly isolated human peripheral blood mononuclear cells (PBMC) are a limited resource, but are in high demand for hematological and immunological research^1–3^. The Trima Accel apheresis device (Terumo BCT, Inc., Lakewood, USA) for collecting platelets incorporates a leukocyte reduction system (LRS) to minimize contamination of thrombocyte products with leukocytes (Fig. 1a). PBMC captured in LRS chambers as a by-product of platelet donation have long been exploited in research^3–5^ as a source of cells from healthy donors because of their convenience, cell quality and pathogen-free origin (Fig. 1b). From 2023 onwards, the device manufacturer mandated an update of the running software from Trima Accel version 6 to version 7. According to the manufacturer, this update implemented an improved algorithm for platelet collection and LRS management with interface regulation to achieve a shorter processing time and higher platelet yields. The main advantage of the new software version is to increase the proportion of donors who are qualified for double platelet collection due to higher productivity^6^. In general, the manufacturer recommends the use of platelet additive solution (PAS) instead of autologous plasma as a suspension medium to minimize plasma mediated risks like platelet transfusion reactions^7^ (Fig. 1c). PAS like Intersol and T-PAS are nutritive solutions containing citrate, acetates, phosphates and salts. PAS is added after the collection procedure to the platelet concentrates for stabilization during storage, as well as avoiding transfusion of large plasma volumes to patients and improving photochemical inactivation of pathogens in platelet concentrates^8^. The effects of PAS on platelets has been investigated in several in vitro and in vivo studies^9^. Platelet concentrates collected with Trima 7 platelet-in-PAS showed less residual leukocytes than those collected in plasma^10^. Long-term donation using apheresis devices with LRS chambers has been associated with T cell lymphopenia^11–13^ that some studies, but not others^14^, associate an increased risk of infections^15^. To mitigate loss of lymphocytes, a so-called “rinseback” procedure can be inserted at the end of the apheresis process to flush cells back to the donor^16^.

The modifications introduced by Trima Accel version 7 potentially impacted researchers who relied upon cells collected using version 6 for their research. Hence, the aim of our study was to document software-dependent differences in cellular composition of LRS chamber concentrates, and to investigate the influence of using PAS instead of plasma.

## Results

### LRS chamber concentrates differ between Trima 6 and 7

**Fig. 2:**
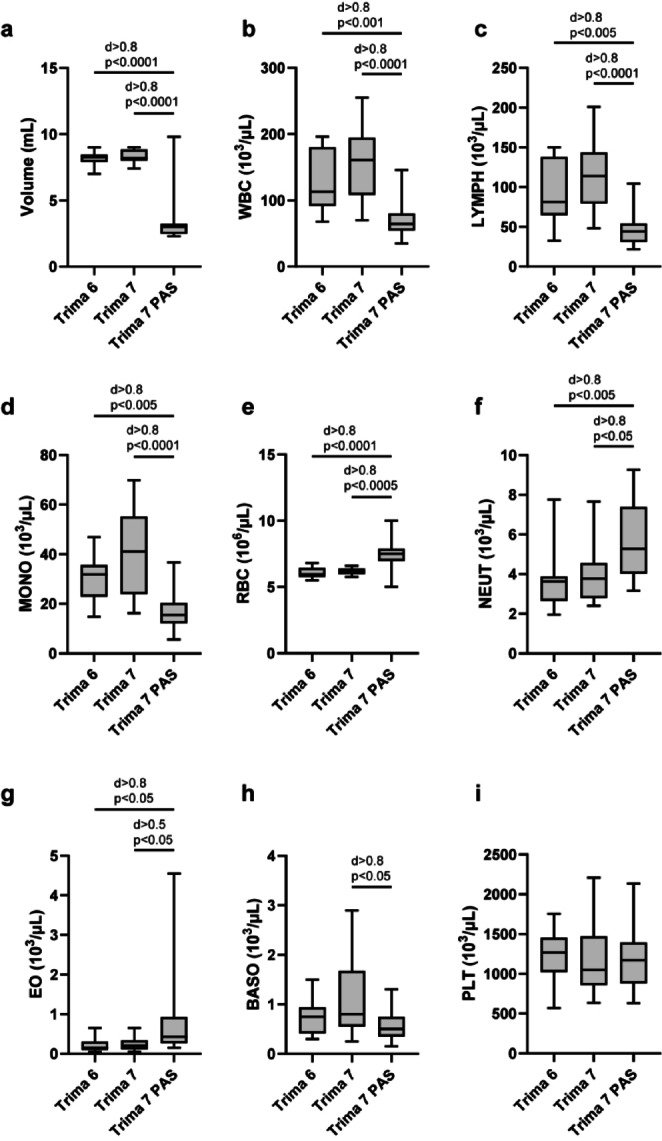
After platelet apheresis with Trima 7 and platelet additive solution substantial differences in cellular composition of LRS chamber products were observed. Leukocyte product was recovered from LRS chamber and total volume was determined (**a**). The cellular composition was analyzed, including white blood cell (**b**), lymphocytes (**c**), monocytes (**d**), red blood cells (**e**), neutrophils (**f**), eosinophils (**g**), basophils (**h**) and platelets (**i**) (n = 16 for Trima 6, n = 12 for Trima 7 and n = 20 for Trima 7 PAS; for (**a**) it is n = 13 for Trima 7 and n = 14 for Trima 7 PAS; statistical analyses were carried out using one-way ANOVA with Tukey test for multiple comparisons; effect size is reported as Cohen’s d).

LRS chambers were collected after platelet apheresis running with Trima Accel software version 6 using plasma (Trima 6), Trima Accel software version 7 using plasma (Trima 7) and Trima 7 using platelet additive solution (Trima 7 PAS). The volume of concentrate was recorded and its cellular composition was determined using an automated Sysmex cell counter. Trima 7 PAS yielded only 3.4 ± 1.9 mL concentrate, compared to Trima 6 (8.1 ± 0.5 mL, p < 0.0001, d > 0.8) and Trima 7 (8.3 ± 0.5 mL, p < 0.0001, d > 0.8) (Fig. 2a). White blood cell (WBC) concentrations were also decreased after apheresis with Trima 7 PAS (71.0 ± 27.8 × 10^3^/µL) compared to Trima 6 (125.8 ± 43.4 × 10^3^/µL, p < 0.001, d > 0.8) and Trima 7 (161.4 ± 55.8 × 10^3^/µL, p < 0.0001, d > 0.8) (Fig. 2b). Of note, Trima 7 PAS products contained significantly lower concentrations of lymphocytes (Fig. 2c) and monocytes (Fig. 2d) than Trima 6 or Trima 7 apheresates. In contrast, using PAS resulted in more erythrocytes (Fig. 2e), neutrophils (Fig. 2f) and eosinophils (Fig. 2g). Basophil concentrations differed between Trima 7 and Trima 7 PAS (Fig. 2h). Platelet concentrations were comparable for all three conditions (Fig. 2i). Overall, the yield of PBMC (lymphocytes, monocytes and basophils) from Trima 7 PAS LRS chambers was significantly lower in absolute (Table S1) and relative terms (Fig. S1), especially after adjusting for recovered concentrate volumes (Fig. S2).

### T cell subset distribution is affected by Trima 7

**Fig. 3:**
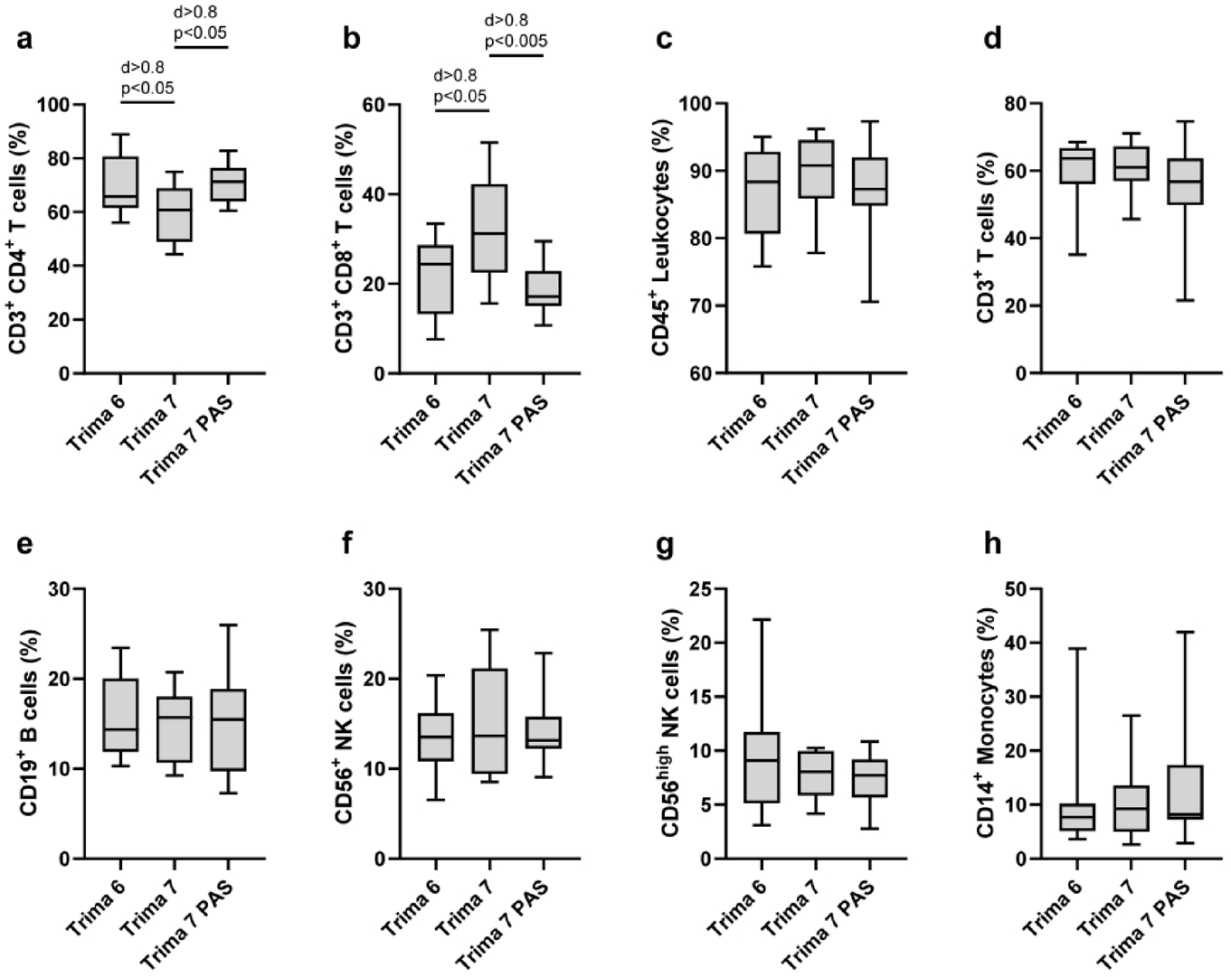
Flow cytometry analysis of LRS chamber cell products revealed differences in CD4^+^ and CD8^+^ T cell frequencies. Leukocyte product was recovered from LRS chamber and stained for flow cytometry analysis. Frequencies of cell populations were determined by manual gating, including CD3^+^ CD4^+^ T cells (**a**), CD3^+^ CD8^+^ T cells (**b**), CD45^+^ leukocytes (**c**), CD3^+^ T cells (**d**), CD19^+^ B cells (**e**), CD56^+^ NK cells (**f**), CD56^high^ NK cells (**g**) and CD14^+^ monocytes (**h**) (n = 15 for Trima 6, n = 9 for Trima 7 and n = 14 for Trima 7 PAS; statistical analyses were carried out using one-way ANOVA with Tukey test for multiple comparison; effect size is reported as Cohen’s d).

LRS chamber concentrates were characterized by flow cytometry and leukocyte subset frequencies were extracted by manual gating (Fig. S3). Compared to Trima 6 (69.9 ± 10.7%, p < 0.05, d > 0.8) and Trima 7 PAS (70.8 ± 7.7%, p < 0.05, d > 0.8) the frequency of CD4^+^ T cells was significantly lower in Trima 7 concentrates (59.6 ± 10.8%) (Fig. 3a). Frequencies of CD8^+^ T cells were correspondingly increased in Trima 7 (32.0 ± 11.7%) compared to Trima 6 (22.1 ± 8.6%, p < 0.05, d > 0.8) and Trima 7 PAS (18.7 ± 5.4%, p < 0.005, d > 0.8) (Fig. 3b). No other significant differences in frequencies of manually gated populations were found (Fig. 3c–h; Table S2).

**Fig. 4:**
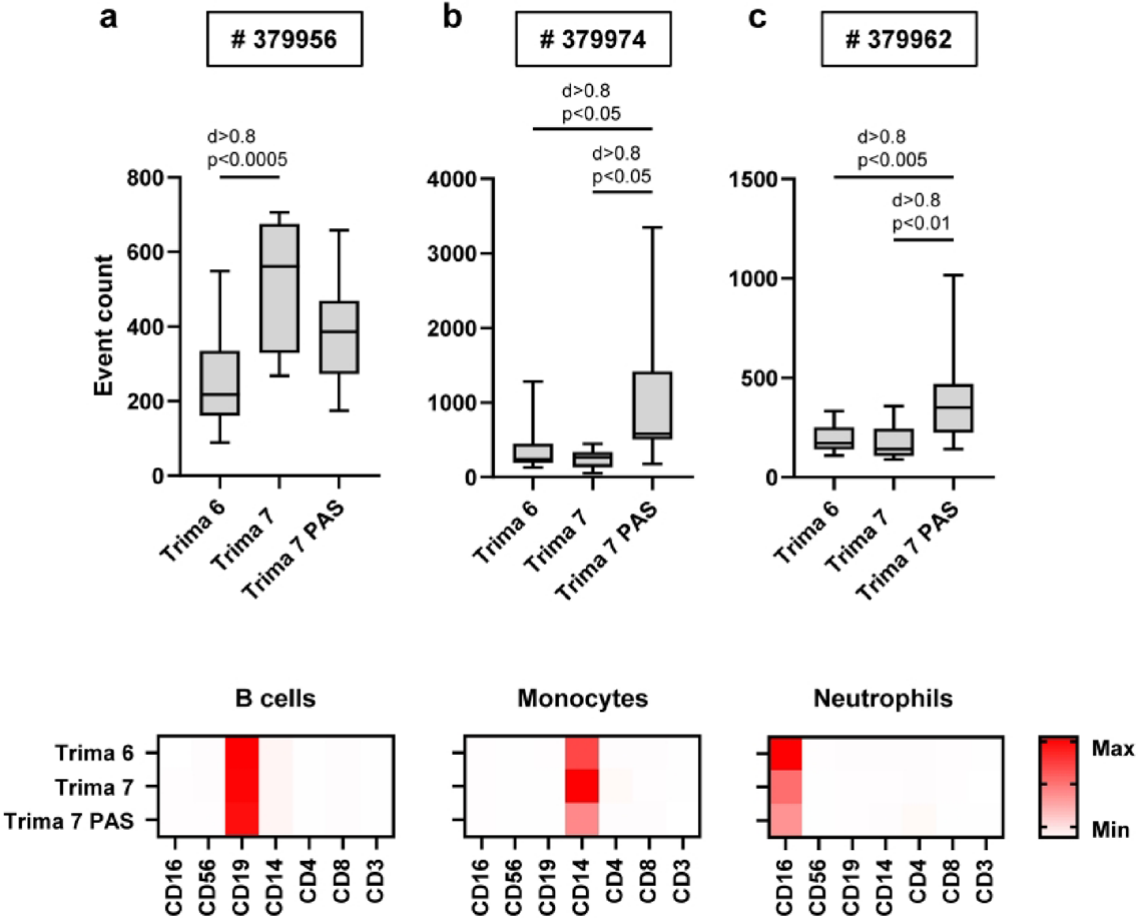
CITRUS analysis of flow cytometry data revealed non-T cell apheresis-dependent differences. Flow cytometry data were fully recompensated and uploaded into Cytobank. CITRUS analysis was performed using PAMR as predictive model. Absolute numbers of events were determined in the three non-T cell metaclusters (**a**) # 379956, (**b**) # 379974 and (**c**) # 379962. (n = 15 for Trima 6, n = 9 for Trima 7 and n = 14 for Trima 7 PAS; statistical analyses were carried out using one-way ANOVA with Tukey test for multiple comparisons; effect size is reported as Cohen’s d). Heatmaps were generated displaying MFI per channel to phenotypically characterize the selected clusters (one representative sample per condition is shown as an example).

CITRUS is a machine learning algorithm for analyzing flow cytometry data^17,18^. CITRUS defined 38 cell clusters and 12 metaclusters showing biological signatures between Trima 6, Trima 7 and Trima 7 PAS samples (Fig. S4). Exploring the phenotype of these cell subsets revealed that 9 of 12 metaclusters represented T cell subpopulations, including eight CD4^+^ T cell subsets and one CD8^+^ T cell subset (Fig. S5). The remaining three metaclusters lacked T cell markers, but expressed CD16, CD19 or CD14. To explore differences between sample groups, we compared absolute numbers of events per cluster, confirming differences in manually gated data — namely, a Trima 7-dependent increase in CD8^+^ T cells (Fig. S6a) and a corresponding decrease in CD4^+^ T cells (Fig. S6b). Furthermore, CITRUS was able to find additional apheresis-dependent differences in CD4^+^ cell clusters that were not identified by manual gating (Fig. S6 c-i). The three non-T cell metaclusters comprised CD19^+^ B cells (Fig. 4a), CD14^+^ monocytes (Fig. 4b) and CD16^+^ neutrophils (Fig. 4c).

## Discussion

Here, we report that upgrading from Trima 6 to Trima 7 software only slightly affects the composition of leukocyte concentrates from Trima Accel LRS chambers when plasma was used as suspension medium. In contrast, recovered volume and cell yield were greatly reduced when using PAS and a rinseback with plasma at the end of the apheresis process. Researchers using PBMC from LRS chambers should be aware that changes to the cell collection protocol impact the cells present in their starting material and also possibly influence their function.

Using an automated cell counter to analyze the cellular composition of LRS chambers produced with Trima 6 or 7, we found higher numbers of all leucocytes compared to some previously published data^3,19^. Such variability likely reflects local variations in clinical protocols and instrument settings; for instance, differences in anticoagulation and flow management. No gross differences were found in concentrates produced with Trima 6 or 7 using plasma as the suspension medium using the Sysmex counter. However, flow cytometry did suggest software-dependent differences between Trima 6 and 7. Whereas proportion of total CD3^+^ cells remained unchanged, differences in the composition of CD4^+^ and CD8^+^ T cell subpopulations were observed. We attribute this difference to a donor sampling effect in our small cohort so that differences are due to donor-to-donor variation, and not to the mechanics of cell collection. Irradiating platelet products prevents proliferation of CD8^+^ T cells, so reduces GvHD risk^20^. If collection conditions reduced the number of residual CD8^+^ T cells in platelet products, we might expect a clinical benefit in lowering GvHD risk. Further work is necessary to assess whether CD8^+^ T cells numbers are consistently reduced in PAS versus plasma products.

When running apheresis with the updated software version 7 and PAS as suspension medium we found lower numbers of lymphocytes and monocytes. This may result from implementing a rinseback with plasma at the end of the collection process with Trima 7 PAS. This additional step intends to mitigate lymphodepletion of the donor avoiding a higher susceptibility for infections. Additionally, the software programmed for collection with PAS may also influence the numbers of lymphocytes and monocytes in the LRS chamber as reducing them possibly during the centrifugation step of apheresis or during the rinseback due to their lower density compared to granulocytes^21^.

Flow cytometry is a powerful tool for analyzing a wide range of biological markers within one single blood sample but the downstream analysis is limited by the restriction of manual gating in particular when working with high dimensional data. Machine learning algorithms like CITRUS can be a promising approach for the analysis of complex single cell datasets by identification of stratifying biological signatures between different treatment groups^17,18^. Here, CITRUS analysis of LRS chamber flow cytometry data not only confirmed results obtained by automated cell counting or manual gating but also revealed new software-dependent differences in B cell populations not discovered by manual gating so far. Furthermore, CITRUS was capable to identify additional CD4^+^ T cell clusters showing apheresis-dependent biological signatures that were not be detected by manual gating. Computational approaches like CITRUS are known to be superior to manual gating in terms of efficiency, reproducibility and reduction of human bias^22^. In contrast to manual gating, the CITRUS algorithm combines all samples into one aggregate dataset before identifying cell populations by hierarchical clustering of phenotypically similar cells. Cells are then assigned back to the individual samples and cluster characteristics are calculated^23^.

In research routine, PBMC isolated from LRS chamber product serve as source for the isolation of monocytes^24–26^ that are often further differentiated into macrophages or dendritic cells^4,5,27,28^. So far, our data focusses on cellular composition of concentrates and does not allow any statement to be made about activation status or functionality of recovered cells. These important aspects need to be addressed in further investigations.

Here, we report for the first time that apheresis procedure with PAS and plasma rinseback influences the composition of leukocytes that are enriched in the leukocyte reduction system. Potential confounders due to different donor characteristics as age, sex and individual peripheral blood cell distribution may influence the composition of LRS products. However, we attribute the observed differences to the technical aspects of the collection process when running a platelet-in-PAS apheresis.

In conclusion, we found only subtle differences between concentrates obtained with Trima 6 versus Trima 7 software when using plasma as the suspension medium. However, using PAS instead of plasma led to a much reduced cell yield and grossly altered leukocyte subset distributions. Thus, researchers have to be aware of the LRS chamber origin and have to take in account whether apheresis was run with plasma or PAS. We highlight this issue so others can assess the impact of software update and suspension medium in their own experimental systems.

## Methods

### Apheresis procedure and sampling protocol

Apheresis from healthy platelet donors were performed by the Department of Transfusion Medicine at University Hospital Regensburg using the Trima Accel automated blood collection system (Terumo BCT, Inc., Lakewood, USA). The device ran either Trima Accel software version 6 or version 7 with plasma for platelet collection. Platelet apheresis was run with an ACDA ratio of 1:10 and 3.5–4.5 L processed blood volume. Per donor a maximum of 26 plateletpheresis per year is allowed^14^. LRS chamber products generated during apheresis with software version 7 and PAS (Intersol or T-PAS +) were obtained from external suppliers (Blutspendedienst BRK GmbH, Regensburg, Germany or ITM Suhl gGmbH, Suhl, Germany). Here, a rinseback with plasma was inserted at the end of the apheresis process. This study was authorized by the local ethics committee (approval 22–2780-101) and all participants gave full, informed written consent. Samples were collected from APR-2023 until MAY-2024.

### Automated analysis of LRS chamber products

Leukocyte concentrates were recovered from LRS chambers and total volume was determined using a serological pipette. Concentrates were diluted with PBS (1:5) and their cellular composition was analyzed using an automated cell counter (XN-550, Sysmex, Norderstedt, Germany) returning values for white blood cells (WBC), red blood cells (RBC), platelets (PLT), lymphocytes (LYMPH), monocytes (MONO), neutrophils (NEUT), eosinophils (EO) and basophils (BASO).

### Clinical flow cytometry

Cell concentrates were stained for flow cytometry using the DURAClone IM Phenotyping Basic Tube (B53328, Beckman Coulter, Krefeld, Germany) according to standard operating procedures^29–31^. Data were recorded with a Navios cytometer running Cytometry List Mode Data Acquisition and Analysis Software version 1.3 (Beckman Coulter). Analyses were performed using Kaluza software version 2.2 (Beckman Coulter).

### Statistics and visualisation

Data visualisation and statistical analyses were performed using GraphPad Prism version 10.3.1 (GraphPad Software, Inc., Boston, USA). Statistics were calculated using one-way ANOVA with Tukey test for multiple comparison. P-values were given in categories as p < 0.05, p < 0.01, p < 0.005, p < 0.001, p < 0.0005 and p < 0.0001. Effect size was calculated in SPSS version 29 (IBM SPSS Statistics, New York, USA). Cohen’s d was reported in categories (d > 0.2, d > 0.5 and d > 0.8).

### CITRUS analysis

For the identification of stratifying biological signatures in a complex single cell dataset, multi-parameter flow cytometry data of LRS chamber products were analyzed using CITRUS algorithm available on Cytobank Premium platform (Beckman Coulter Life Sciences, Indianapolis, USA). Data were recompensated and analyzed in Kaluza (software version 2.2, Beckman Coulter) according to the described gating strategy (Fig. S3). Compensated events were exported through the CD45^+^ leukocyte gate and uploaded into Cytobank. After scaling and assigning the data to the corresponding treatment groups (Trima 6, Trima 7 and Trima 7 PAS), a CITRUS analysis was run using PAMR as predictive model with a minimum cluster size of 0.5%, a tenfold cross validation and a false discovery rate of 1.0%. Event counts were exported for all metaclusters and statistically analyzed and visualized using GraphPad Prism (software version 10.3.1).

## Electronic supplementary material

Below is the link to the electronic supplementary material.

Supplementary Material 1

## Data Availability

Data are available from the corresponding author upon reasonable request.
