# Supplementary material for: Platelet apheresis with additive solution and plasma rinseback affects the cellular composition of LRS chamber products
Source: Sci Rep. 2025 Jun 6;15:19923. doi: 10.1038/s41598-025-04350-4 (PMC12144167; doi:10.1038/s41598-025-04350-4)
Supplement: Supplementary file 1 — Supplementary Material 1 [file 41598_2025_4350_MOESM1_ESM.pdf]

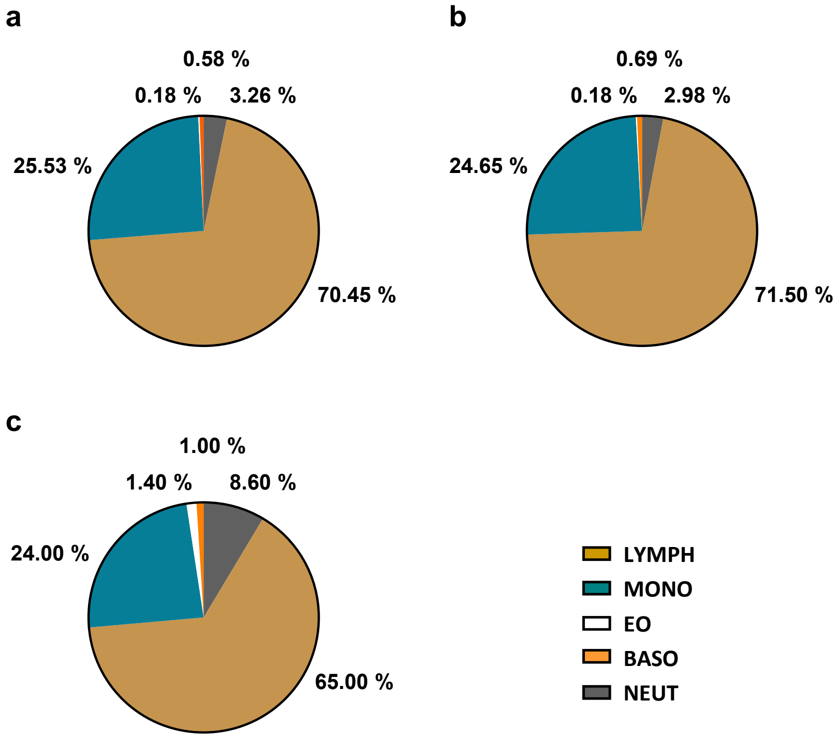

Supplementary Figure 1 *After platelet apheresis with Trima 7 and platelet additive solution LRS chamber products contained higher proportion of granulocytes*. LRS chambers were obtained from platelet apheresis using (a) software Trima Accel version 6 (Trima 6), (b) version 7 (Trima 7) or (c) version 7 with platelet additive solution (Trima 7 PAS). The cellular composition was analysed including neutrophils, lymphocytes, monocytes, eosinophils and basophils (n=16 for Trima 6, n=12 for Trima 7 and n=20 for Trima 7 PAS; mean frequencies of cell populations are shown in % of total).

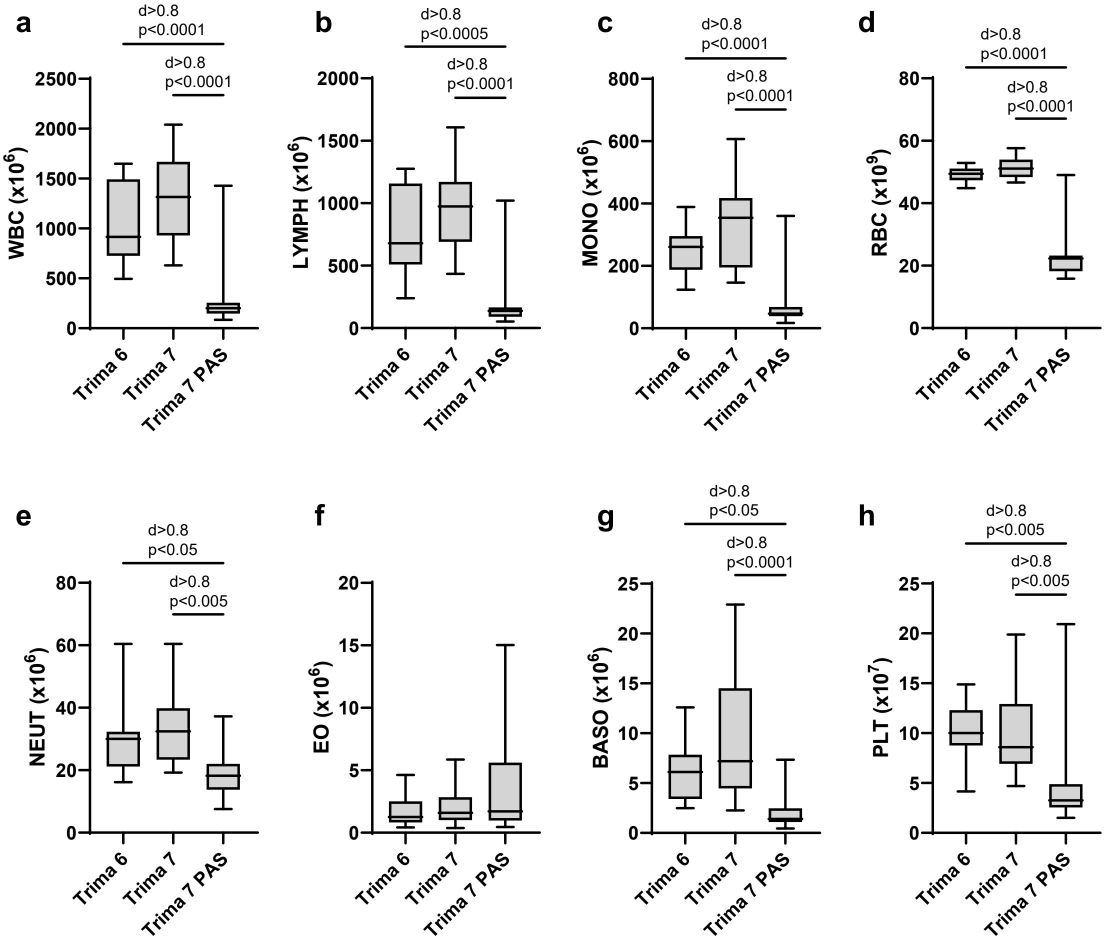

Supplementary Figure 2 *After platelet apheresis with Trima 7 and platelet additive solution substantial differences in absolute cell numbers of LRS chamber products were observed*. Leukocyte product was recovered from LRS chamber and the cellular composition was analyzed. Absolute cell numbers were calculated, including white blood cells (a), lymphocytes (b), monocytes (c), red blood cells (d), neutrophils (e), eosinophils (f), basophils (g) and platelets (h) (n=16 for Trima 6, n=12 for Trima 7 and n=14 for Trima 7 PAS; statistical analyses were carried out using one-way ANOVA with Tukey test for multiple comparisons; effect size is reported as Cohen‘s d).

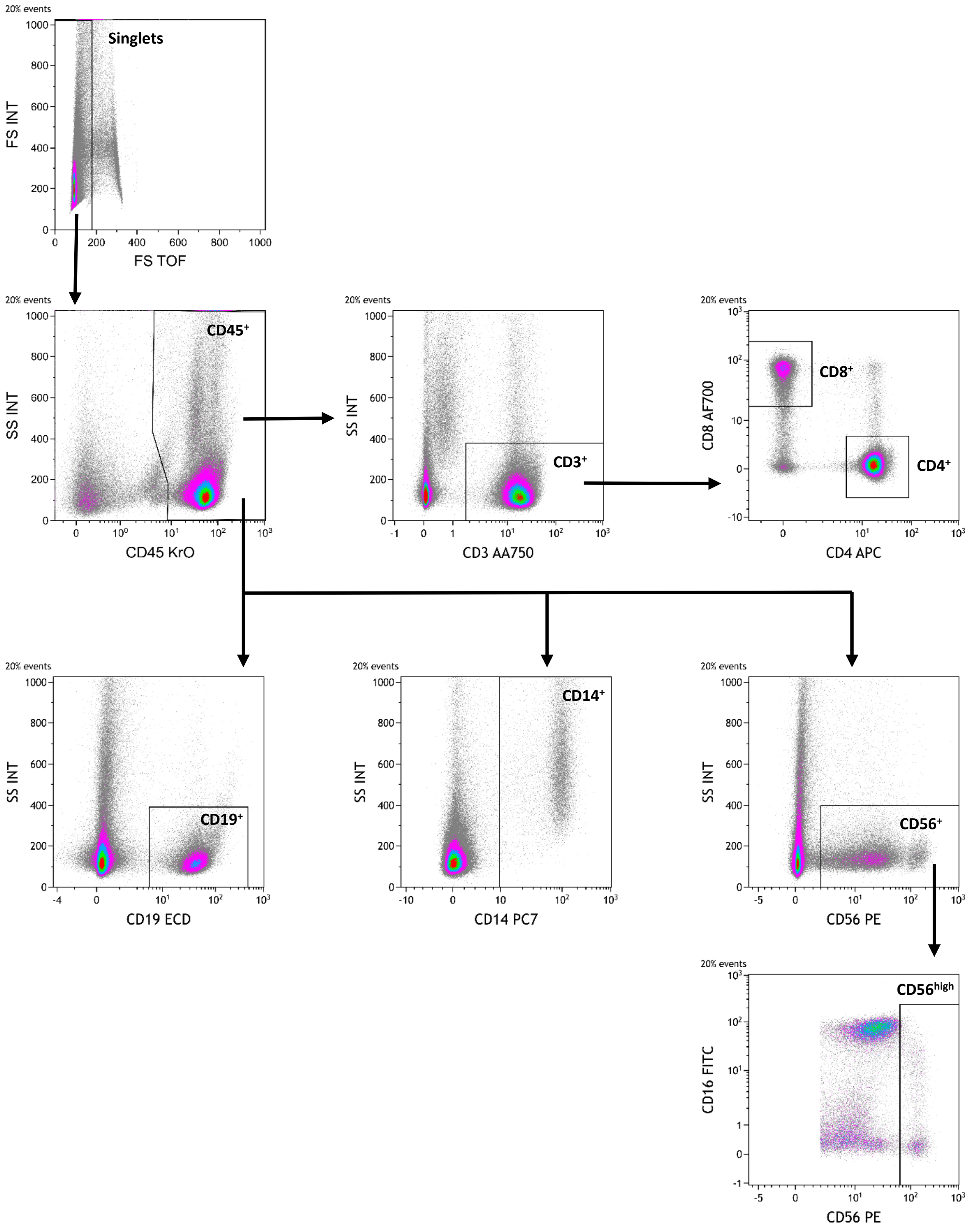

Supplementary Figure 3 *Gating strategy for the DURAClone IM Phenotyping Basic Tube*. LRS chamber leukocyte product was stained for flow cytometry analysis using the dried-down DURAClone IM Phenotyping Basic Tube from Beckman Coulter. Data were analyzed as follows: Selection of singlets; gating on $CD45^{+}$ leukocytes; gating on $CD3^{+}$ T cells; gating on $CD4^{+}$ and $CD8^{+}$ T cells within the $CD3^{+}$ compartment; within $CD45^{+}$ leukocytes gating on $CD19^{+}$ B cells, $CD14^{+}$ monocytes and $CD56^{+}$ NK cells; gating ond $CD56^{high}$ NK cells within the $CD56^{+}$ compartment (one represantative sample is shown as an example).

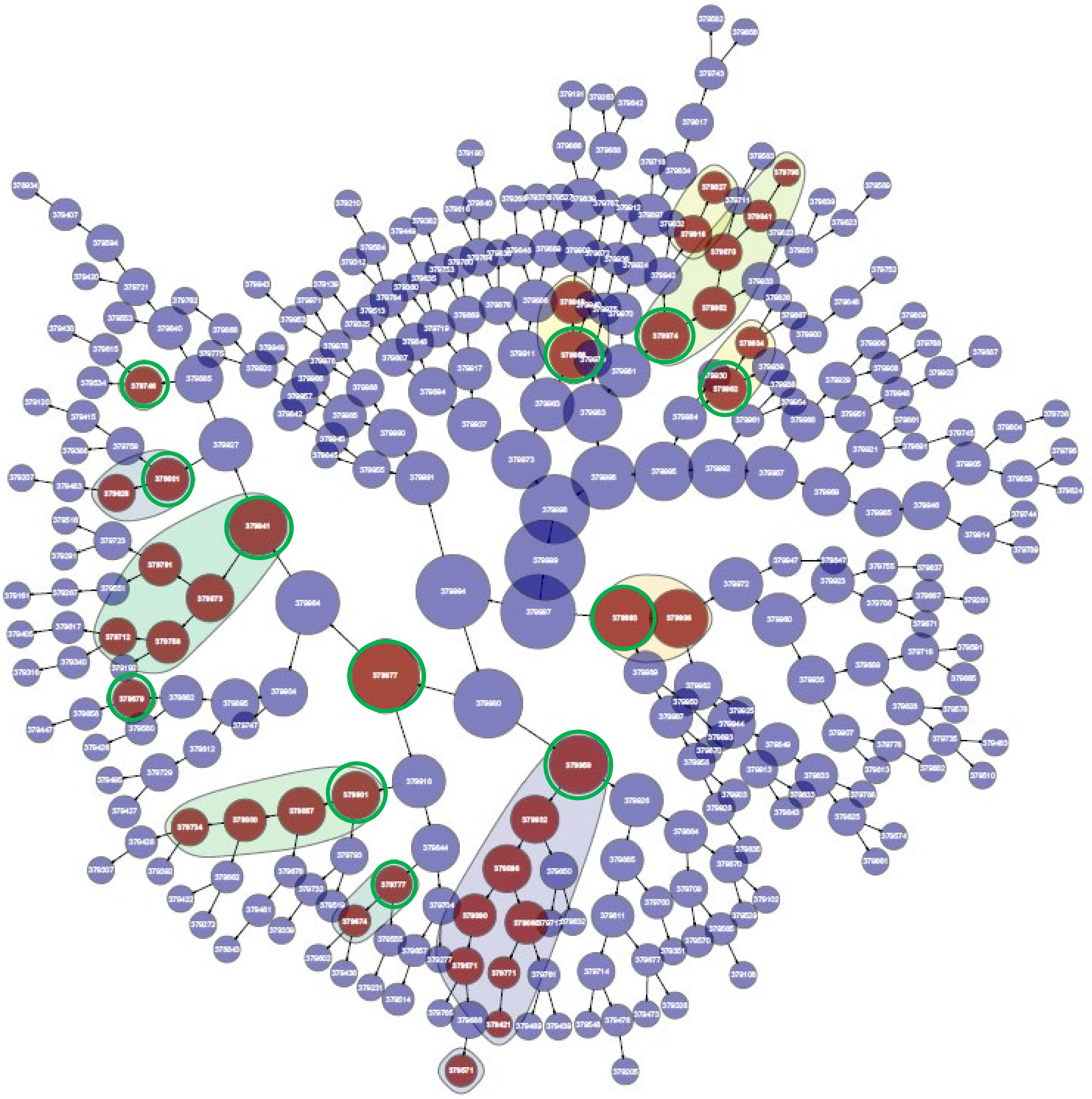

Supplementary Figure 4 *During CITRUS analysis of flow cytometry data phenotypically similiar cells were clustered in nodes in a hierarchical tree*. Flow cytometry data were fully recompensated and uploaded into Cytobank. CITRUS analysis was performed using PAMR as predictive model resulting in 38 cell clusters (nodes in red) and 12 metaclusters (green circles) showing stratifying signatures.

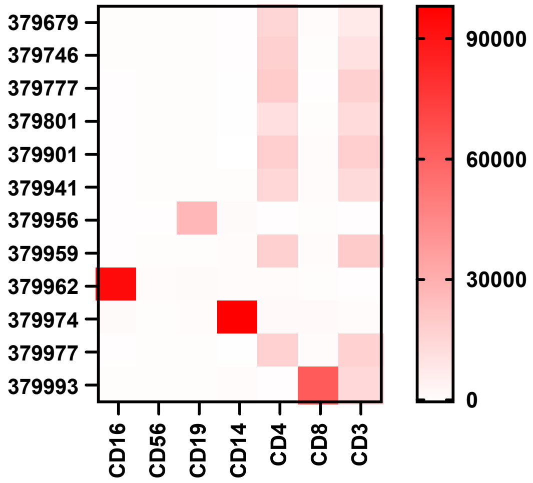

Supplementary Figure 5 *Most CITRUS metaclusters contain T cell subpopulations*. The 12 CITRUS metaclusters were selected and the phenotype of cells was determined by analyzing the MFI for each channel and cluster. Results were displayed as heatmap (one representative sample is shown as an example).

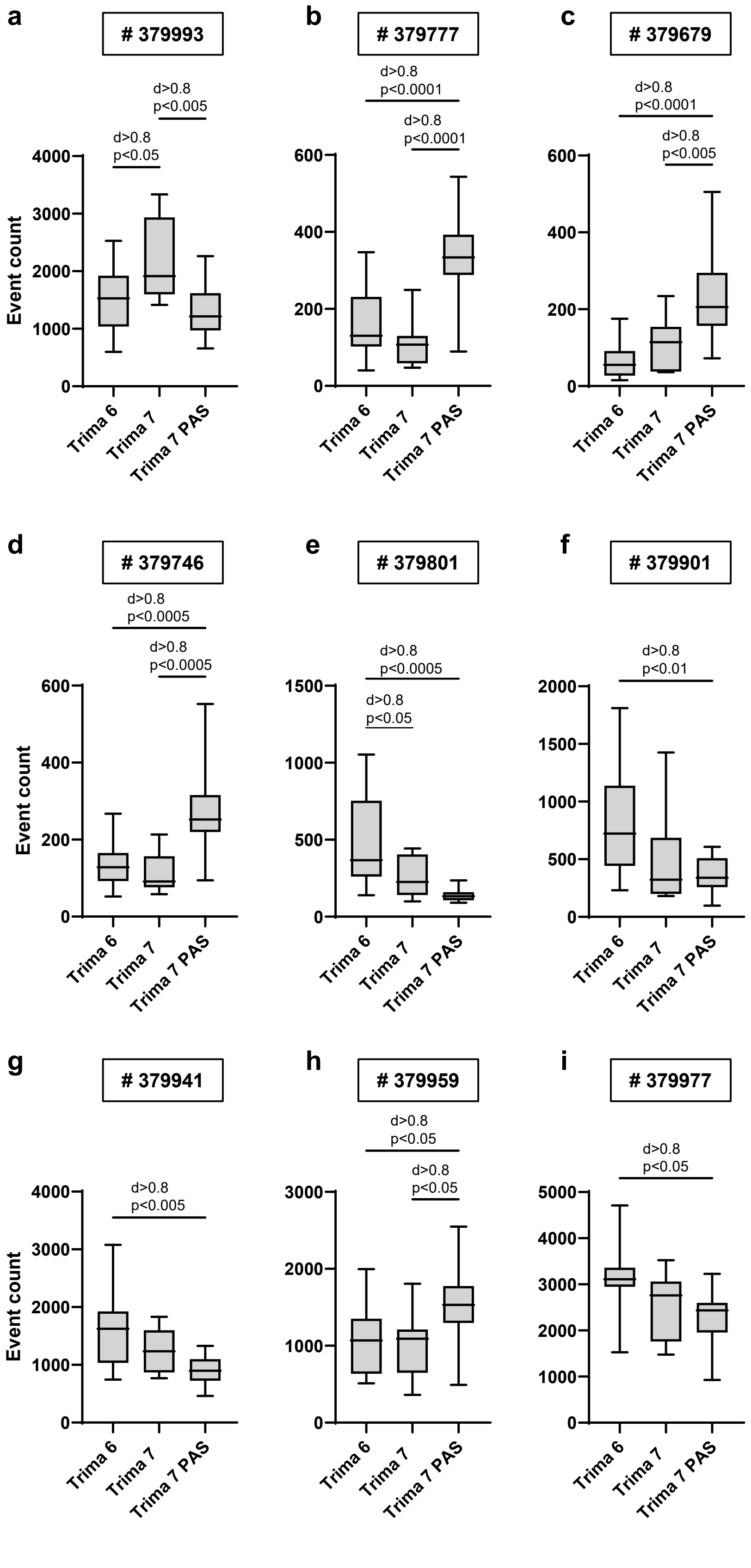

Supplementary Figure 6 *9 of 12 CITRUS metaclusters contain T cell subpopulations.* Leukocyte product was recovered from LRS chamber and stained for flow cytometry analysis. Fully recompensated data were uploaded into Cytobank and a CITRUS analysis was perfomed resulting in 12 metaclusters showing significant signatures between apheresis conditions. In one metacluster biological signatures were based on $CD8^+$ T cells (a). In 8 metacluster differences were based on $CD4^+$ T cell populations (b-i) (n=15 for Trima 6, n=9 for Trima 7 and n=14 for Trima 7 PAS; statistical analyses were carried out using one-way ANOVA with Tukey test for multiple comparisons; effect size is reported as Cohen‘s d). The three remaining metaclusters not shown here revealed differences in $CD19^+$, $CD14^+$ and $CD16^+$ cell populations (Fig. 4).

		Mean	SD	Min	Max	N
Volume (ml)	Trima 6	8,14	0,49	7,00	9,00	16
	Trima 7	8,30	0,50	7,40	9,00	13
	Trima 7 AS	3,39	1,89	2,30	9,80	14
WBC (10^3/µl)	Trima 6	125,80	43,42	67,65	196,10	16
	Trima 7	161,40	55,77	70,05	255,10	12
	Trima 7 AS	71,01	27,79	34,95	145,70	20
LYMPH (10^3/µl)	Trima 6	90,79	39,13	32,65	150,00	16
	Trima 7	116,30	46,42	48,20	200,90	12
	Trima 7 AS	46,87	21,22	21,75	104,20	20
MONO (10^3/µl)	Trima 6	30,44	8,83	14,75	46,90	16
	Trima 7	39,55	17,49	16,25	69,80	12
	Trima 7 AS	16,90	7,47	5,66	36,75	20
RBC (10^6/µl)	Trima 6	6,06	0,39	5,50	6,80	16
	Trima 7	6,18	0,27	5,75	6,60	12
	Trima 7 AS	7,40	1,12	5,00	10,00	20
NEUT (10^3/µl)	Trima 6	3,65	1,37	1,95	7,75	16
	Trima 7	4,12	1,67	2,40	7,65	12
	Trima 7 AS	5,67	1,89	3,15	9,25	20
EO (10^3/µl)	Trima 6	0,22	0,17	0,05	0,65	16
	Trima 7	0,25	0,19	0,05	0,65	12
	Trima 7 AS	0,96	1,20	0,15	4,55	20
BASO (10^3/µl)	Trima 6	0,73	0,32	0,30	1,50	16
	Trima 7	1,12	0,77	0,25	2,90	12
	Trima 7 AS	0,60	0,32	0,15	1,30	20
PLT (10^3/µl)	Trima 6	1243,00	314,80	570,00	1755,00	16
	Trima 7	1206,00	466,10	635,00	2210,00	12
	Trima 7 AS	1204,00	375,50	630,00	2135,00	20

Supplementary Table 1 *Detailed values of LRS chamber cell populations analyzed using an automated cell counter*. LRS chambers were obtained from platelet apheresis using software Trima Accel version 6 (Trima 6), version 7 (Trima 7) or version 7 with additive solution (Trima 7 AS). Using a Sysmex Cell Counter the cellular composition of LRS chamber product was analyzed. Results are reported as mean, standard deviation (SD), minimum (Min), maximum (Max) and number of values (N).

		Mean	SD	Min	Max	N
CD45⁺ Leukocytes (%)	Trima 6	86,91	6,57	75,83	95,09	15
	Trima 7	89,91	6,06	77,83	96,22	9
	Trima 7 AS	87,03	7,44	70,60	97,30	14
CD3⁺ T cells (%)	Trima 6	59,42	10,51	35,11	68,55	15
	Trima 7	60,84	7,86	45,68	71,18	9
	Trima 7 AS	55,58	12,88	21,57	74,63	14
CD3⁺ CD4⁺ T cells (%)	Trima 6	69,90	10,73	56,14	88,92	15
	Trima 7	59,58	10,75	44,26	74,92	9
	Trima 7 AS	70,78	7,65	60,48	82,82	14
CD3⁺ CD8⁺ T cells (%)	Trima 6	22,11	8,55	7,64	33,41	15
	Trima 7	32,01	11,68	15,65	51,51	9
	Trima 7 AS	18,65	5,39	10,72	29,47	14
CD19⁺ B cells (%)	Trima 6	15,71	4,41	10,29	23,43	15
	Trima 7	14,81	4,01	9,23	20,73	9
	Trima 7 AS	15,42	5,51	7,26	25,99	14
CD56⁺ NK cells (%)	Trima 6	13,44	4,14	6,53	20,35	15
	Trima 7	14,98	6,21	8,53	25,43	9
	Trima 7 AS	14,03	3,39	9,07	22,85	14
$CD56^{high}$ NK cells (%)	Trima 6	9,38	5,60	3,10	22,16	15
	Trima 7	7,80	2,20	4,18	10,25	9
	Trima 7 AS	7,38	2,44	2,71	10,87	14
CD14⁺ Monocytes (%)	Trima 6	11,02	10,51	3,59	38,95	15
	Trima 7	10,27	7,43	2,61	26,54	9
	Trima 7 AS	12,23	10,13	2,86	41,98	14

Supplementary Table 2 *Detailed results of cell population frequencies analyzed by manual gating of flow cytometry data*. LRS chambers were obtained from platelet apheresis using software Trima Accel version 6 (Trima 6), version 7 (Trima 7) or version 7 with additive solution (Trima 7 AS). Using the dried-down DURAClone IM Phenotyping Basic Tube (Beckman Coulter) LRS chamber leukocytes were stained for flow cytometry analysis. Frequencies of cell populations (in %) were determined by manual gating according to gating strategy described in Supplementary Figure 4. Results are reported as mean, standard deviation (SD), minimum (Min), maximum (Max) and number of values (N).
